# Three-Dimensional Modeling and Performance Analysis of Dynamic mmWave V2I Networks Based on Stochastic Geometry †

**DOI:** 10.3390/s26123963

**Published:** 2026-06-22

**Authors:** Hui Zheng, Haocheng Yang, Peng Wu

**Affiliations:** 1 School of Information and Communication Engineering, Beijing Information Science and Technology University, Beijing 100192, China; zhenghui@bistu.edu.cn (H.Z.); haocheng.yang@bistu.edu.cn (H.Y.); 2 National Innovation Institute of Defense Technology, Academy of Military Science, Beijing 100071, China

**Keywords:** stochastic geometry, vehicular network, high mobility, 3D antenna array, beam alignment

## Abstract

Millimeter-wave (mmWave) technology is essential for meeting the reliable connectivity and high-capacity demands of autonomous driving applications. Vehicle-to-infrastructure (V2I) networks have been modeled and analyzed based on stochastic geometry (SG) in many studies. However, most studies focus only on two-dimensional (2D) antenna models and disregard a key characteristic of V2I networks, i.e., the rapid mobility of vehicles. In this work, a three-dimensional (3D) coverage and connectivity analysis framework is proposed for mmWave V2I downlink transmission based on SG. First, a realistic 3D system model is developed, which includes 3D transmission channel, blockage, and antenna array models. Then, exact expressions for the coverage probability, connectivity probability, and effective throughput of a typical vehicle are derived. Finally, the theoretical analysis is validated through simulation results, which also reveal that an optimal density of roadside units (RSUs) that maximizes spectral efficiency exists and that disregarding the effect of the vertical beam of a 3D antenna array can lead to inaccurate evaluations. Moreover, appropriately setting system parameters can mitigate the negative impact of high vehicular mobility on connectivity performance.

## 1. Introduction

Benefiting from the rapid progress of artificial intelligence (AI), autonomous vehicles (AVs) are envisioned to enable a safer, more efficient, and greener intelligent transportation system (ITS) [1,2,3]. To realize advanced ITS use cases, AVs require various sensors (e.g., LiDAR, cameras, ultrasound, and infrared sensors) to frequently collect surrounding traffic information and communicate with roadside units (RSUs) [4,5,6]. These applications require strict reliability, very low latency, and ultra-high data rates, which cannot be supported by current vehicular communication systems [7,8,9]. According to Third-Generation Partnership Project (3GPP) standards, millimeter-wave (mmWave) technology was designed to fulfill the reliable connectivity and high-capacity demands of autonomous driving applications [10,11]. On the one hand, mmWave communication technology provides an abundant amount of available bandwidth; on the other hand, it enables the deployment of large-scale yet very compact antennas on vehicles, thereby achieving directional transmission and mitigating adjacent interference [12,13]. Due to these characteristics, mmWave vehicle-to-infrastructure (V2I) networks have been considered for supporting advanced ITS applications.

In an ITS, vehicles can move at speeds of up to 120–150 km/h. This high mobility results in frequent beam and cell handovers, as well as potential connection interruptions in vehicular networks due to mmWave beam misalignment [14,15]. To mitigate the negative effects of vehicular mobility, some works [16,17,18,19] have proposed advanced beam management methods and network architectures that integrate mmWave and sub-6 GHz frequency bands. However, network performance has typically been evaluated using complicated field trials or simulations, which cannot directly clarify the relationships between key system parameters and performance. To the best of our knowledge, no existing works have analytically studied the impact of vehicular mobility on the three-dimensional (3D) coverage and connectivity performance of mmWave V2I networks. Therefore, establishing an analytical 3D system model for mmWave V2I networks and developing an accurate method for evaluating the impact of mobility-related system parameters on network performance remain open research challenges.

### 1.1. Related Works and Motivations

In recent years, many studies [16,17,18,19] have focused on enhancing the connectivity performance of mmWave vehicular networks considering the high mobility of vehicles. Zhong et al. [16] proposed an image-based beam-tracking method, using deep learning and vehicular environmental information, to improve the probability of beam alignment and the connectivity performance of mmWave V2I networks. By exploiting the regularity of vehicular mobility, Ding et al. [17] proposed a context-aware, standard-compatible beam update scheme for efficient beam alignment in mmWave V2I networks. Kim et al. [18] presented a beam-switching scheme for mmWave vehicle-to-vehicle (V2V)- and V2I-integrated vehicular networks and developed a real-world testbed to demonstrate the transmission performance gain. Zhao et al. [19] explored a dual-connectivity V2I network framework integrating sub-6 GHz and mmWave frequency bands and introduced a predictive beam-tracking method based on machine learning to improve spectrum efficiency. We note that the above works conducted complex field trials or simulations to evaluate network performance, at a high cost in terms of resources and time.

As a powerful mathematical tool, stochastic geometry (SG) has been applied to the system modeling and performance analysis of mmWave V2I networks [20,21,22,23,24]. Tassi et al. [20] developed a theoretical framework to characterize the outage probability of mmWave V2I downlinks in highway scenarios, considering the blockage effects caused by large vehicles with two-dimensional (2D) beamforming. Wang et al. [21] derived the coverage probability of an urban mmWave V2I downlink, considering the Manhattan path loss model. Yi et al. [22] created a tractable spatial model for vehicular platoon systems with the aid of a Matérn hard-core process and introduced V2V links as a backup to enhance the connectivity probability of mmWave vehicular networks. In [23], the authors proposed a relay strategy based on information value for mmWave V2I downlink transmission and analyzed the effects of non-cooperative behavior on coverage probability. Choi et al. investigated the problem of location-correlated blockages among mmWave V2I links in [24] and utilized roadside reconfigurable intelligent surfaces (RISs) to enhance coverage probability in [25]. However, the common drawback of these works is that the effect of vehicle mobility is neglected, which inevitably leads to an inaccurate performance evaluation.

For dynamic mmWave V2I networks, Giordani et al. [26] used SG to analyze the throughput and connectivity probability in a highway scenario with multiple lanes. Bafqi et al. [27] investigated the impact of vehicle trajectory, mobility, and cache schemes on the connectivity probability and transmission delay of mmWave vehicular networks. Aghashahi et al. [28] analyzed the effective connectivity time of mmWave V2I networks, considering beam management and handover. The primary limitation of the studies in [26,27,28] is their focus solely on 2D directional transmission, disregarding the vertical beam control of 3D antenna arrays installed on RSUs or vehicles. However, the vertical dimension is crucial and should not be overlooked, especially considering the short-range transmission characteristic of mmWave communication. Furthermore, compared to 2D beamforming, 3D beamforming offers greater transmission gain and better interference control. In [29], the authors proposed an analytical method for evaluating the connectivity probability of terahertz transmission, considering 3D beamforming but disregarding user mobility. For dynamic mmWave vehicular communication, 3D beamforming significantly increases the complexity of network performance analysis because of the necessity to compute the 3D beam sojourn probabilities and times for a typical vehicle. To the best of our knowledge, this is the first attempt to analyze the coverage and connectivity performance of mmWave V2I networks, considering both high vehicular mobility and 3D beamforming.

### 1.2. Contributions

The contributions of this work can be summarized as follows:

1. A 3D mmWave V2I system model accounting for blockage, antenna, and channel models is constructed in a unidirectional multi-lane highway environment. To track the rapid mobility of vehicles and maintain the high quality of mmWave V2I connections, we adopt the periodic beam alignment scheme proposed by the 3GPP [17,30] to correct beam misalignment periodically after the initial beam acquisition.
2. An analytical and tractable framework is established based on SG to compute the coverage probability, connectivity probability, and effective throughput. In particular, the impact of high vehicular mobility and 3D beamforming is addressed by exploiting the geometric relationships between transceivers to analyze the horizontal and vertical beam sojourn probabilities and times. Moreover, simplified analytical results of the coverage and connectivity performance under 2D beamforming are also obtained, including closed-form expressions in special cases.
3. Extensive simulations are conducted to demonstrate the proposed analysis methods. The simulation results indicate that an optimal RSU density that maximizes the spectral efficiency exists, which decreases as vehicle speed increases. Moreover, disregarding the effects of vertical beams leads to inaccurate evaluations of coverage and connectivity performance, particularly in scenarios with frequent beam alignment. More importantly, the negative impact of high vehicular mobility on the connectivity performance of mmWave V2I networks can be mitigated by appropriately setting the RSU density, beam alignment period, and beamwidth.

The remainder of this work is organized as follows. Section 2 introduces the mmWave V2I communication system model, and Section 3 analyzes coverage and connectivity performance. The simulation results of the performance evaluation are shown in Section 4, and Section 5 summarizes the findings of this work.

## 2. System Model

### 2.1. Network Model

**Figure 1:**
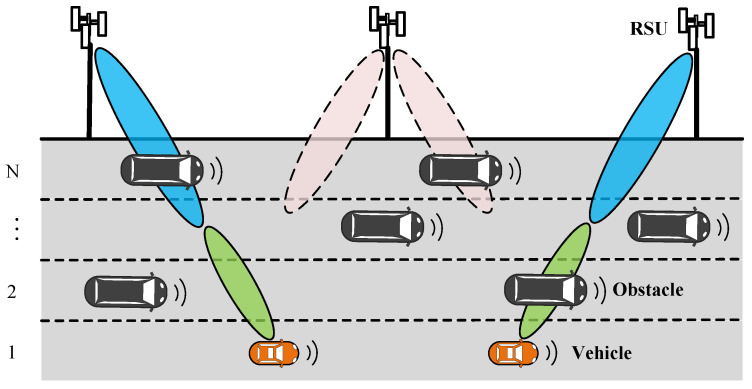
Representation of 3D mmWave V2I downlink transmission system model.

A unidirectional *N*-lane road segment is shown in Figure 1, where the length of each lane is infinite and the width is *W*. The mmWave transceiver-equipped RSUs are positioned adjacent to the outermost lane at a distance of 0.5 W from the road edge. The least favorable situation is considered: small target vehicles (cars) move along the innermost roadway, and large blocking vehicles (such as trucks and buses), which can block V2I transmission from RSUs to target vehicles, travel in N−1 outer lanes [22]. For clarity, large blocking vehicles are hereafter referred to as “obstacles”. Additionally, it is assumed that all vehicles and obstacles maintain a constant speed (*v*) while moving in a straight line.

In this work, the positions of vehicles and RSUs are modeled as one-dimensional (1D) homogeneous Poisson point processes (HPPPs), denoted as $\Phi_{U}$ and $\Phi_{B}$, with respective densities of $\lambda_{u}$ and $\lambda_{b}$. The positions of obstacles are similarly characterized by N−1 independent 1D HPPPs with a density of $\lambda_{k}$. For simplicity and to mitigate boundary effects, the typical vehicle $u_{0}$ is located at the origin of the innermost lane.

### 2.2. Channel and Blockage Models

Due to the high penetration loss of mmWave [31,32], the impact of non-line-of-sight (NLOS) signals is considered negligible. As in [23], the line-of-sight (LOS) channel is given by

(1) $$L\left(d\right)=\mu d^{-\rho },$$

where ρ and μ represent the path loss parameters and *d* denotes the 3D distance from the typical vehicle $u_{0}$ to its serving RSU $b_{0}$, expressed as follows:

(2) $$d=\sqrt{r^{2}+y^{2}+h^{2}}.$$

**Figure 2:**
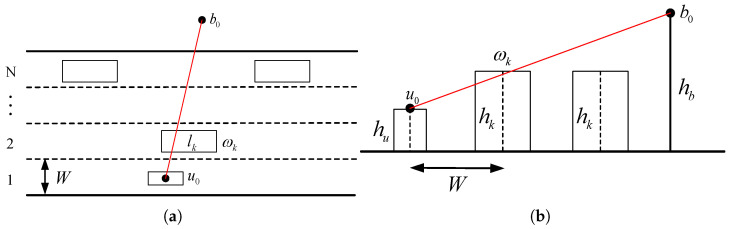
Representation of 3D obstacle model. (**a**) Top view. (**b**) Side view.

In this context, *r*, *y*, and *h* represent the 3D distance projections of *d*. The obstacle is modeled as a cuboid, and its 3D size is denoted by $l_{k}$, $w_{k}$, and $h_{k}$, as depicted in Figure 2. Note that $h_{b}$ and $h_{u}$ are the heights of RSUs and vehicles, respectively. Hence, we have $h=h_{b}-h_{u}$ and y=NW.

### 2.3. Three-Dimensional Beamforming

**Figure 3:**
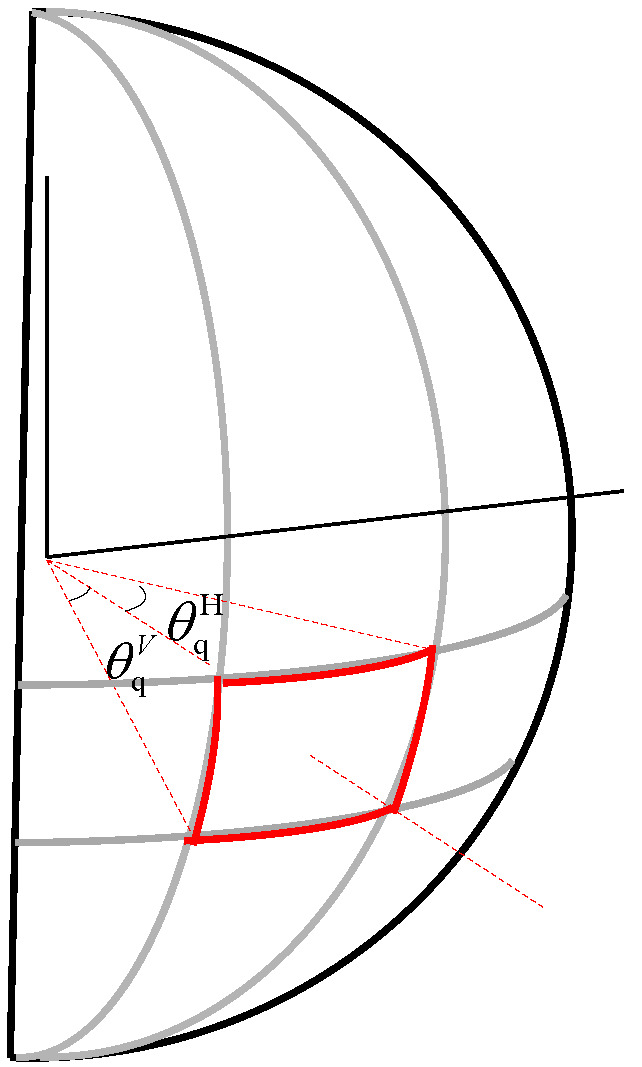
Representation of 3D antenna radiation model.

As illustrated in Figure 3, we propose a 3D pyramidal antenna array model, where $\theta_{q}^{H}$ and $\theta_{q}^{V}$ represent the horizontal and vertical beamwidths, respectively, with the subscript q∈{b,u} distinguishing between RSUs and vehicles. For the antenna array, the formulation of the main lobe gain is [33]

(3) $$G_{q}=\frac{\pi }{\arcsin\left(\tan\left(\frac{\theta_{q}^{H}}{2}\right)\tan\left(\frac{\theta_{q}^{V}}{2}\right)\right)}.$$

Under high mobility conditions, the beam alignment procedure is crucial for maintaining directional beam gain and continuous connectivity between transceivers. According to 3GPP reports [17,30], the beam alignment process is triggered periodically or by the received signal strength falling below a given threshold. However, how to choose the appropriate beam period or threshold has not yet been determined. For simplicity, we assume that the transceivers’ beams are perfectly aligned at the beginning of every beam alignment period $t_{s}$. Subsequently, $b_{0}$ maintains the same beamforming configuration within a period, and $u_{0}$ adaptively aligns its receiving beam according to its location. If $u_{0}$ moves out of the coverage area of $b_{0}$ within a period, misalignment and connectivity interruption occur [26,27]. In such instances, connectivity can only be reestablished at the beginning of the next period.

### 2.4. Transmission Signal

This work assumes that each vehicle connects to its nearest RSU with identical bandwidth *B*. Moreover, the interfering link’s beam direction is modeled as a uniformly distributed random variable ranging from 0 to π. Our analysis focuses only on interfering RSUs that satisfy the following conditions: they have a line-of-sight (LOS) path to $u_{0}$; their primary beams cover $u_{0}$; and they are simultaneously within the coverage of $u_{0}$’s main beam.

According to the aforementioned models, the received signal at $u_{0}$ is given by

(4) $$\begin{array}{cc} Y & =\sqrt{PG_{b}G_{u}L\left(d\right)}X+\sum_{i\in \Phi_{B_{0}}\setminus \left\{b_{0}\right\}}\sqrt{PG_{b}G_{u}L\left(d_{i}\right)}X_{i}+Z, \end{array}$$

where *X* is the desired signal received from $b_{0}$, $X_{i}$ represents the interfering signal from the *i*-th RSU $b_{i}$, and $d_{i}$ denotes the distance between $b_{i}$ and $u_{0}$. $G_{b}$ and $G_{u}$ are the main lobe gains of the antennas at the RSUs and vehicles, respectively. *Z* is the noise with power $\sigma^{2}$, *P* is the transmission power, and $\Phi_{B_{0}}$ denotes the set of RSUs that introduce interference at $u_{0}$. It is assumed that all interfering signals are independently and identically distributed (i.i.d.).

Based on Equation (4), the received signal-to-interference-plus-noise ratio (SINR) at $u_{0}$ is expressed as

(5) $$\mathrm{SINR}=\frac{L(d)}{I_{d}+\sigma^{2}/\left(PG_{b}G_{u}\right)},$$

where $I_{d}$ refers to the normalized total interference power, given by

(6) $$I_{d}=\sum_{i\in \Phi_{B_{0}}\setminus \left\{b_{0}\right\}}L\left(d_{i}\right).$$

## 3. Performance Analysis

This section characterizes the coverage, connectivity, and throughput performance of mmWave V2I downlink communication using SG. First, the LOS probability of $u_{0}$ under the multi-lane highway scenario is obtained. Then, expressions for the coverage probability, connectivity probability, and effective throughput of $u_{0}$ are derived, considering high vehicular mobility and 3D beamforming. The results under 2D beamforming are also derived for comparative evaluation.

### 3.1. LOS Probability

As shown in Figure 2, the minimum obstacle height in the *n*-th lane (2≤n≤N) that potentially blocks the LOS link between an RSU and a vehicle is denoted by $H_{n}$. This implies that the LOS link can be obstructed by an obstacle in the *n*-th lane when satisfying the condition $H_{n}<h_{k}$. Using the principles of similar triangles, we obtain

(7) $$\frac{h_{b}-h_{u}}{H_{n}-h_{u}}=\frac{WN}{\left(n-1\right)W-0.5w_{k}},$$

where

(8) $$H_{n}=\frac{2W\left(h_{u}\left(N-n+1\right)+h_{b}\left(n-1\right)\right)-w_{k}\left(h_{b}-h_{u}\right)}{2WN}.$$

Consequently, the overall number of blocked lanes is expressed as

(9) $$N_{b}=\mathrm{cardinality}\left\{\underset{2\leq n\leq N}{⋃}\left\{n:H_{n}<h_{k}\right\}\right\}.$$

**Lemma** **1.** *The probability of a LOS connection for the V2I link between $u_{0}$ and $b_{0}$ is expressed as*

(10) $$P_{L}=\exp\left(-\lambda_{k}l_{k}N_{B}\right).$$

**Proof.**  For V2I communication between $u_{0}$ and $b_{0}$, the LOS transmission is established when there are no obstacles within $l_{k}/2$ on both sides of the V2I link. According to the assumption that obstacles are independently distributed across various blockage lanes [34], the LOS probability can be expressed as

(11) $$P_{L}=\exp{\left(-\lambda_{k}l_{k}\right)}^{N_{B}}=\exp\left(-\lambda_{k}l_{k}N_{B}\right).$$

□

Lemma 1 indicates that $P_{L}$ is inversely proportional to the obstacle density $\lambda_{k}$ and obstacle length $l_{k}$, and that it is independent of the RSU density $\lambda_{b}$ and vehicle density $\lambda_{u}$.

### 3.2. Coverage Probability

The coverage probability $P_{\mathrm{cov}}$ is defined as the probability that the SINR received at $u_{0}$ is greater than a given threshold *T*. Hence, we have $P_{\mathrm{cov}}\left(T\right)=P\left[\mathrm{SINR}>T\right]$. To simplify the interference calculation, the predominant interference analysis method is adopted according to [35,36], which focuses exclusively on the interferer that causes interruptions at the receiver.

**Lemma** **2.** *The expression for the coverage probability of $u_{0}$ under T is*

(12) $$\begin{array}{c} P_{\mathrm{cov}}\left(T\right)=2\lambda_{b}P_{L}\times \left(\int_{0}^{\tau }e^{-2\lambda_{b}r-\beta_{1}\kappa_{1}\left(T,r\right)}dr+\int_{\tau }^{\infty }e^{-2\lambda_{b}r-\beta_{1}\kappa_{2}\left(T,r\right)}dr\right), \end{array}$$

*where*

(13) $$\begin{array}{c} \kappa_{1}\left(z_{1},z_{2}\right)=\lambda_{b}P_{L}\left(\min\left\{J\left(z_{1},z_{2}\right),J\left(z_{2}\right)\right\}-r\right), \end{array}$$

(14) $$\begin{array}{c} \kappa_{2}\left(z_{1},z_{2}\right)=\lambda_{b}P_{L}\left(J\left(z_{1},z_{2}\right)-r\right), \end{array}$$

(15) $$\begin{array}{c} J\left(z\right)=\sqrt{{\left(\frac{h\left(\sqrt{z^{2}+y^{2}}+h\tan\left(\frac{\theta_{u}^{V}}{2}\right)\right)}{h-\sqrt{z^{2}+y^{2}}\tan\left(\frac{\theta_{u}^{V}}{2}\right)}\right)}^{2}-y^{2}}, \end{array}$$

(16) $$\begin{array}{c} J\left(z_{1},z_{2}\right)=\sqrt{{\left(\frac{{\left(z_{2}^{2}+y^{2}+h^{2}\right)}^{-\frac{\rho }{2}}}{z_{1}}-\frac{\sigma^{2}}{P\mu G_{b}G_{u}}\right)}^{-\frac{2}{\rho }}-y^{2}-h^{2}}, \end{array}$$

*and $\tau =\sqrt{\frac{h^{2}}{\tan^{2}\left(\frac{\theta_{u}^{V}}{2}\right)}-y^{2}}$, $\beta_{1}=\frac{2\theta_{b}^{H}\theta_{u}^{H}\theta_{b}^{V}}{\pi^{3}}$.*

**Proof.**  The proof is provided in Appendix C in [23]. □

Note that the result in (12) contains only a single integral, which is independent of the vehicle speed (*v*). Moreover, $P_{\mathrm{cov}}$ decreases with increasing *T* and $\lambda_{k}$. If $\lambda_{k}\to \infty$, we have $P_{\mathrm{cov}}\to 0$.

### 3.3. Connectivity Probability

The connectivity probability, denoted by $P_{C}$, is defined as the probability that the following two conditions are satisfied simultaneously:

1. The SINR received at $u_{0}$ is larger than *T* at the beginning of the beam alignment period;
2. $u_{0}$ stays in the transmit beam range of $b_{0}$ during the entire beam alignment period, i.e., it maintains beam alignment.

If either of these conditions is not satisfied, the target V2I transmission link between $u_{0}$ and $b_{0}$ is interrupted. Obviously, the probability of meeting condition (1) is $P_{\mathrm{cov}}$, as given in (12). The probability of meeting condition (2) is denoted by the beam sojourn probability $P_{B}$ [27], expressed as $P_{B}=P\left[R>vt_{s}\right]$, with *R* denoting the distance from $u_{0}$ to the beam coverage edge of $b_{0}$ in the direction of $u_{0}$’s movement. Therefore, the connectivity probability $P_{C}$ is given by $P_{C}\left(T\right)=P_{\mathrm{cov}}\left(T\right)\;\cdot \;P_{B}$. It should be noted that $P_{B}=1$ if, and only if, $u_{0}$ remains within the coverage of both the vertical and horizontal beams of $b_{0}$ throughout the beam alignment period.

**Theorem** **1.** *The expression for the connectivity probability of $u_{0}$ under T is*

(17) $$\begin{array}{cc} P_{C}\left(T\right)=\lambda_{b}P_{L} & \times (I\left(A_{1}\right)\int_{r_{k_{1}}}^{\tau }e^{-2\lambda_{b}r-\beta_{1}\kappa_{1}\left(T,r\right)}dr+\int_{c_{1}}^{\infty }e^{-2\lambda_{b}r-\beta_{1}\kappa_{2}\left(T,r\right)}dr\\ \; & +I\left(A_{2}\right)\int_{r_{k_{2}}}^{\tau }e^{-2\lambda_{b}r-\beta_{1}\kappa_{1}\left(T,r\right)}dr+\int_{c_{2}}^{\infty }e^{-2\lambda_{b}r-\beta_{1}\kappa_{2}\left(T,r\right)}dr), \end{array}$$

*where*

(18) $$\begin{array}{c} r_{k_{1},k_{2}}^{H}=\sqrt{\frac{v^{2}t_{s}^{2}}{2}+\frac{yvt_{s}}{\tan(\frac{\theta_{b}^{H}}{2})}\pm \frac{v^{2}t_{s}^{2}}{2}\sqrt{1+\frac{4y}{vt_{s}\tan\left(\frac{\theta_{b}^{H}}{2}\right)}-\frac{4y^{2}}{v^{2}t_{s}^{2}}}-y^{2}}, \end{array}$$

(19) $$\begin{array}{c} r_{k_{1},k_{2}}^{V}=\sqrt{\frac{v^{2}t_{s}^{2}}{2}+\frac{hvt_{s}}{\tan(\frac{\theta_{b}^{V}}{2})}\pm \frac{v^{2}t_{s}^{2}}{2}\sqrt{1+\frac{4h}{vt_{s}\tan\left(\frac{\theta_{b}^{V}}{2}\right)}-\frac{4h^{2}}{v^{2}t_{s}^{2}}}-h^{2}}. \end{array}$$

*where I(x) is the indicator function; $r_{k_{1}}=\max\left(r_{k_{1}}^{H},r_{k_{1}}^{V}\right)$; $r_{k_{2}}=\max\left(r_{k_{2}}^{H},r_{k_{2}}^{V}\right)$; $A_{1}=r_{k_{1}}<\tau$; $A_{2}=r_{k_{2}}<\tau$; $c_{1}=\max\left(r_{k_{1}},\tau \right)$; and $c_{2}=\max\left(r_{k_{2}},\tau \right)$.*

**Proof.**  See Appendix A. □

Note that the result shown in (17) contains only a single integral, which is influenced by a number of factors, including the densities of RSUs ($\lambda_{b}$) and obstacles ($\lambda_{k}$), the speed (*v*) of vehicles, and the beam alignment duration ($t_{s}$). Moreover, $P_{C}$ decreases with increasing $\lambda_{k}$, *v*, and $t_{s}$. In the case of $\lambda_{k}\to \infty$ or v→∞, we have $P_{C}\to 0$.

### 3.4. Effective Throughput

The effective throughput (*Q*) is the average transmission capacity of $u_{0}$ over the entire beam alignment period, i.e.,

(20) $$\begin{array}{c} Q=Q_{\max}\;\cdot \;\frac{t}{t_{s}}, \end{array}$$

where $Q_{\max}$ represents the peak transmission capacity and *t* is the beam sojourn time [27]. Note that *t* depends on both the speed (*v*) of $u_{0}$ and the 3D beam coverage range of $b_{0}$.

**Theorem** **2.** *The expression for the effective throughput of $u_{0}$ is*

(21) $$\begin{array}{c} Q=\frac{t}{t_{s}}\int_{0}^{\infty }P_{\mathrm{cov}}\left(2^{\frac{t}{B}}-1\right)dt_{0}, \end{array}$$

*where*

(22) $$\begin{array}{c} t=P_{S}t_{s}+\frac{1}{v}\int_{0}^{r_{k_{1}}}\lambda_{b}e^{-2\lambda_{b}r}\min\left(d_{1},d_{1}^{\prime }\right)dr+\frac{1}{v}\int_{0}^{r_{k_{2}}}\lambda_{b}e^{-2\lambda_{b}r}\min\left(d_{2},d_{2}^{\prime }\right)dr, \end{array}$$

(23) $$\begin{array}{c} P_{S}=\frac{e^{-2\lambda_{b}r_{k_{1}}}+e^{-2\lambda_{b}r_{k_{2}}}}{2}, \end{array}$$

(24) $$\begin{array}{c} d_{1,2}=\frac{\sqrt{r^{2}+y^{2}}\sin\left(\frac{\theta_{b}^{H}}{2}\right)}{\cos\left(\frac{\theta_{b}^{H}}{2}\right)\frac{y}{\sqrt{r^{2}+y^{2}}}\pm \sin\left(\frac{\theta_{b}^{H}}{2}\right)\sqrt{1-\frac{y^{2}}{r^{2}+y^{2}}}}, \end{array}$$

(25) $$\begin{array}{c} d_{1,2}^{\prime }=\frac{\sqrt{r^{2}+h^{2}}\sin\left(\frac{\theta_{b}^{V}}{2}\right)}{\cos\left(\frac{\theta_{b}^{V}}{2}\right)\frac{h}{\sqrt{r^{2}+h^{2}}}\pm \sin\left(\frac{\theta_{b}^{V}}{2}\right)\sqrt{1-\frac{h^{2}}{r^{2}+h^{2}}}}. \end{array}$$

**Proof.**  The proof is shown in the appendix in [37]. □

Note that the result shown in (21) contains only a single integral, which is influenced by a number of factors, including the density of RSUs ($\lambda_{b}$) and obstacles ($\lambda_{k}$), the speed (*v*) of vehicles, and the beam alignment duration ($t_{s}$). Moreover, *Q* decreases with increasing $\lambda_{k}$, *v* and $t_{s}$. In the case of $\lambda_{k}\to \infty$ or v→∞, we have Q→0.

### 3.5. Analysis Under 2D Beamforming

In order to highlight the influence of 3D beamforming, we formulate expressions for the coverage probability, connectivity probability, and effective throughput under 2D beamforming, disregarding the vertical beam of the antenna array. The 2D beamforming gain is expressed as

(26) $$G_{q}^{\prime }=\frac{\pi }{\arcsin\left(\tan\left(\frac{\theta_{q}^{H}}{2}\right)\right)}.$$

**Corollary** **1.** *For 2D beamforming, the coverage probability in* (12) *is expressed as*

(27) $$\begin{array}{c} P_{\mathrm{cov}}^{\prime }\left(T\right)=2\lambda_{b}P_{L}\int_{0}^{\infty }e^{-2\lambda_{b}r-\beta_{2}\kappa_{3}\left(T,r\right)}dr, \end{array}$$

*where*

(28) $$\begin{array}{c} \kappa_{3}\left(z_{1},z_{2}\right)=2\lambda_{b}P_{L}\left(J^{\prime }\left(z_{1},z_{2}\right)-r\right), \end{array}$$

(29) $$\begin{array}{c} J^{\prime }\left(z_{1},z_{2}\right)=\sqrt{{\left(\frac{{\left(z_{2}^{2}+y^{2}+h^{2}\right)}^{-\frac{\rho }{2}}}{z_{1}}-\frac{\sigma^{2}}{P\mu G_{b}^{\prime }G_{u}^{\prime }}\right)}^{-\frac{2}{\rho }}-y^{2}-h^{2}}, \end{array}$$

*and $\beta_{2}=\frac{\theta_{b}^{H}\theta_{u}^{H}}{\pi^{2}}$.*

**Proof.**  By substituting $P_{3D}=P_{H_{b}}P_{H_{u}}P_{V_{b}}P_{V_{u}}$ with $P_{2D}=P_{H_{b}}P_{H_{u}}$ in the derivation of Lemma 2, (27) is obtained. □

**Corollary** **2.** *For 2D beamforming, the connectivity probability in* (17) *is expressed as*

(30) $$\begin{array}{c} P_{C}^{\prime }\left(T\right)=\lambda_{b}P_{L}\left(\int_{r_{k_{1}}^{H}}^{\infty }e^{-2\lambda_{b}r-\beta_{2}\kappa_{3}\left(T,r\right)}dr+\int_{r_{k_{2}}^{H}}^{\infty }e^{-2\lambda_{b}r-\beta_{2}\kappa_{3}\left(T,r\right)}dr\right). \end{array}$$

**Proof.**  By substituting $P_{\mathrm{cov}}\left(T\right)$ and $P_{B}$ with $P_{\mathrm{cov}}^{\prime }\left(T\right)$ and $P_{B}^{H}$, respectively, in the derivation of Theorem 1 in Appendix A, (30) is obtained. □

**Corollary** **3.** *For 2D beamforming, the effective throughput in* (21) *is expressed as*

(31) $$\begin{array}{c} Q^{\prime }=\frac{t^{\prime }}{t_{s}}\int_{0}^{\infty }2\lambda_{b}P_{L}\int_{0}^{\infty }e^{-2\lambda_{b}r-\beta_{2}\kappa_{3}(2^{\frac{t_{0}}{B}}-1,r)}drdt_{0}, \end{array}$$

*where*

(32) $$\begin{array}{c} t^{\prime }=P_{S}^{\prime }t_{s}+\frac{1}{v}\int_{0}^{r_{k_{1}}^{H}}\lambda_{b}e^{-2\lambda_{b}r}d_{1}dr+\frac{1}{v}\int_{0}^{r_{k_{2}}^{H}}\lambda_{b}e^{-2\lambda_{b}r}d_{2}dr, \end{array}$$

*and*

(33) $$\begin{array}{c} P_{S}^{\prime }=\frac{e^{-2\lambda_{b}r_{k_{1}}^{H}}+e^{-2\lambda_{b}r_{k_{2}}^{H}}}{2}. \end{array}$$

Compared with 3D beamforming, 2D beamforming results in a lower antenna gain, more interfering RSUs, and lower $P_{\mathrm{cov}}$. On the other hand, 2D beamforming may enhance the probability and duration of beam sojourn due to the broader beam coverage area of $b_{0}$. Hence, it is crucial to assess the influence of the antenna array’s vertical beam on $P_{C}$ and *Q*, as shown in the subsequent section.

**Corollary** **4.** *Under the condition of high signal-to-noise ratio (SNR), a closed-form solution for $P_{\mathrm{cov}}$ under 2D beamforming when y=0 and h=0 is given by*

(34) $$\begin{array}{c} P_{\mathrm{cov}}^{\prime }\left(T\right)=\frac{P_{L}}{1+\beta_{2}P_{L}\left(T^{\frac{1}{\rho }}-1\right)}. \end{array}$$

**Corollary** **5.** *Under the condition of high SNR, a closed-form solution for $P_{C}$ under 2D beamforming when y=0 and h=0 is given by*

(35) $$P_{C}^{\prime }\left(T\right)=\frac{P_{L}\left(1+\exp\left(-2\lambda_{b}vt_{s}\left(1+\beta_{2}P_{L}\left(T^{\frac{1}{\rho }}-1\right)\right)\right)\right)}{2+2\beta_{2}P_{L}\left(T^{\frac{1}{\rho }}-1\right)}.$$

Obviously, $P_{\mathrm{cov}}$ in (34) and $P_{C}$ in (35) both increase with decreasing *T* and increasing ρ, and $P_{C}$ can be improved by decreasing *v* and $t_{s}$. Moreover, with increasing $\theta_{b}^{H}$ and $\theta_{u}^{H}$, $P_{\mathrm{cov}}$ is degraded due to lower beamforming gain and larger interference.

## 4. Numerical Results and Discussion

In this section, we first demonstrate the correctness of the above results through Monte Carlo (MC) simulations. Subsequently, the numerical results are used to evaluate the effects of important system parameters, including $\lambda_{b}$, *v*, and $t_{s}$, on the coverage, connectivity, and throughput performance of mmWave V2I downlinks. We introduce a simulation scenario in which a single-direction, four-lane highway with a lane width of 3.5 m and length of 10 km is considered, and each simulation result is obtained from $10^{8}$ realizations. Table 1 presents the default simulation parameters, following [38,39].

Due to the vehicular mobility considered in this paper, this study evaluates the received SINRs and data rates of $u_{0}$ and *t* during each simulation process. Herein, *t* is obtained based on *v* and the distance between $u_{0}$ and the boundary of the beam coverage area of $b_{0}$. When the received SINR of $u_{0}$ falls below the threshold *T* or *t* is smaller than the beam alignment period $t_{s}$, V2I transmission is regarded as experiencing connection interruptions in this simulation process. After numerous simulations, the average coverage and connectivity probabilities, as well as the effective throughput, can be obtained.

### 4.1. Validation of Results

**Figure 4:**
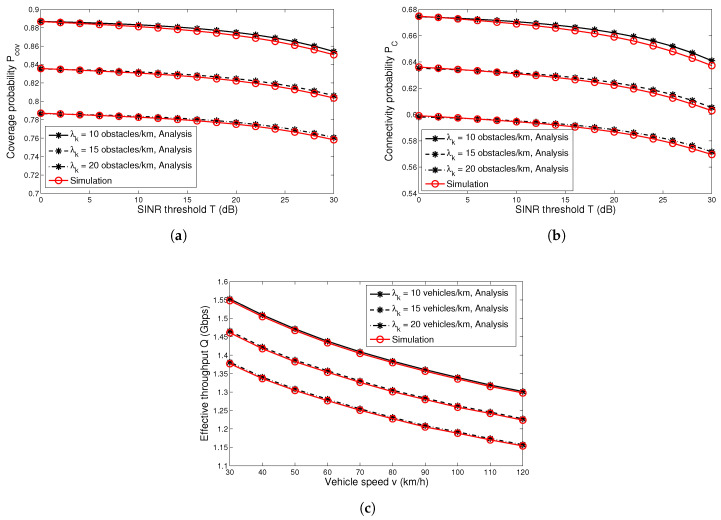
Validation of the proposed framework through Monte Carlo simulations. (**a**) Coverage probability $P_{\mathrm{cov}}$ vs. SINR threshold *T*. (**b**) Connectivity probability $P_{C}$ vs. SINR threshold *T*. (**c**) Effective throughput *Q* vs. vehicle speed *v*.

This section presents the analytical results for $P_{\mathrm{cov}}$, $P_{C}$, and *Q*. Figure 4 compares the analytical results for the coverage probability ($P_{\mathrm{cov}}$), connectivity probability ($P_{C}$), and effective throughput (*Q*) with the MC simulations under various parameter settings. As shown in Figure 4a,b, the analytical results for $P_{\mathrm{cov}}$ and $P_{C}$ closely align with the MC simulations, thereby validating the proposed analytical framework. Since only the predominant interference is considered in the analyses, small discrepancies emerge between the analytical and simulation results, and these discrepancies increase with *T*, leading to underestimation of the total interference and overestimation of $P_{\mathrm{cov}}$ and $P_{C}$. The effect of the interference analysis simplification becomes more pronounced as *T* increases. Notably, the analytical results for *Q* are slightly higher than the simulated results in Figure 4c, providing tight upper bounds. As the obstacle density $\lambda_{k}$ increases, all performance metrics degrade due to a higher number of obstacles obstructing V2I communication.

### 4.2. Numerical Results for $P_{\mathrm{cov}}$

**Figure 5:**
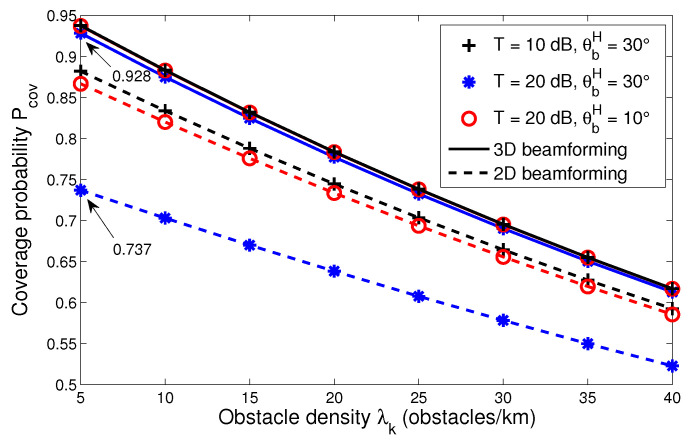
$P_{\mathrm{cov}}$ vs. $\lambda_{k}$ under the two beamforming schemes for various values of *T* and $\theta_{b}^{H}$.

Figure 5 shows the results for $P_{\mathrm{cov}}$ under both 3D and 2D beamforming for various obstacle densities ($\lambda_{k}$), SINR thresholds (*T*), and RSU horizontal beamwidths ($\theta_{b}^{H}$). It is evident that $P_{\mathrm{cov}}$ increases with decreasing $\theta_{b}^{H}$ due to larger beamforming gain and decreased interference from RSUs for $u_{0}$. The performance of $P_{\mathrm{cov}}$ is inferior under simplified 2D beamforming compared to 3D beamforming. This is because the effect of the vertical beam is disregarded, resulting in decreased antenna gain for transceivers and increased interference. Furthermore, the disparity in the performance of $P_{\mathrm{cov}}$ between 3D and 2D beamforming widens as *T* and $\theta_{b}^{H}$ increase.

When T=20 dB and $\theta_{b}^{H}=30$, it is evident that the gap in $P_{\mathrm{cov}}$ between 3D and 2D beamforming is as high as 26%. These results suggest that disregarding the vertical beam may lead to underestimation of coverage performance, especially under high communication thresholds and large beam coverage conditions.

### 4.3. Numerical Results for $P_{C}$

**Figure 6:**
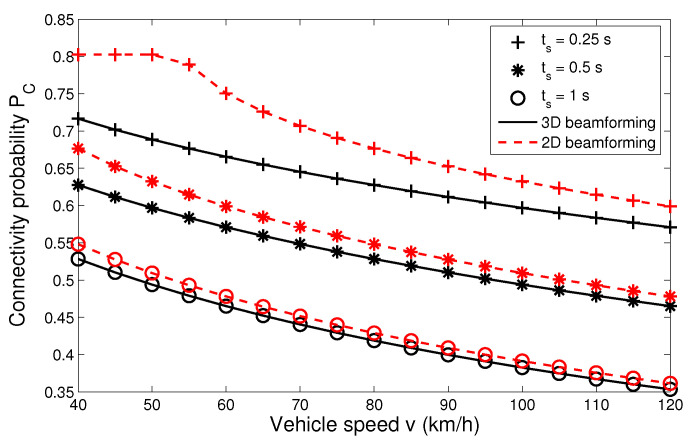
$P_{C}$ vs. *v* under the two beamforming schemes for various values of $t_{s}$.

The results for $P_{C}$ under both 3D and 2D beamforming for various values of *v* and $t_{s}$ are illustrated in Figure 6. Similar to Figure 4c, $P_{C}$ decreases with increasing *v* due to more frequent beam misalignment and a smaller beam sojourn probability $P_{B}$, which is caused by the high mobility of vehicles. Additionally, $P_{C}$ increases with decreasing $t_{s}$ since a smaller $t_{s}$ means more frequent beam alignment, which improves the performance of $P_{B}$. This result reveals that setting a short beam alignment period or a high beam alignment frequency can mitigate the negative impact of vehicular mobility on the connectivity performance of mmWave V2I downlink transmission. Note that frequent beam alignment would cause high signaling overhead, thereby further degrading system performance. Therefore, it is important to select the appropriate beam alignment period, which is left for future work. Furthermore, we note that the result for $P_{C}$ under 2D beamforming is larger than that under 3D beamforming, suggesting that disregarding the vertical beam may lead to overestimation of connectivity performance. This behavior is the opposite to that of $P_{\mathrm{cov}}$, as shown in Figure 5. This is because disregarding the vertical beam leads to not only a decrease in antenna gain and an increase in interference but also less frequent beam misalignment and a higher $P_{B}$. The latter effect is dominant and hence the connectivity probability is overestimated.

**Figure 7:**
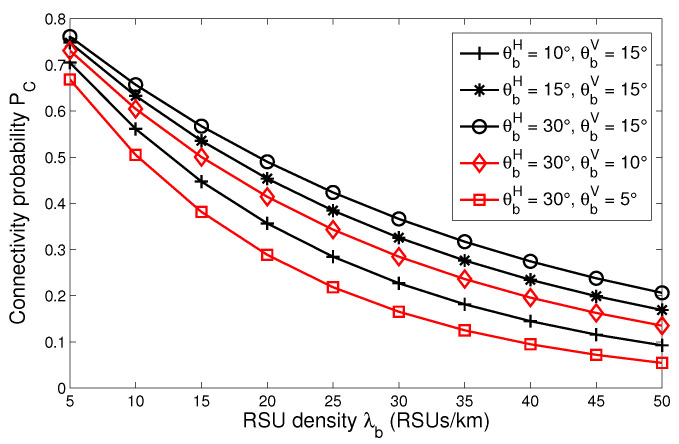
$P_{C}$ vs. $\lambda_{b}$ for various values of $\theta_{b}^{H}$ and $\theta_{b}^{V}$.

Figure 7 illustrates $P_{C}$ vs. the RSU density ($\lambda_{b}$) for various values of the horizontal beamwidth ($\theta_{b}^{H}$) and the vertical beamwidth ($\theta_{b}^{V}$) of the RSUs. It is shown that $P_{C}$ decreases with increasing $\lambda_{b}$ since the densification of RSUs not only introduces more co-channel interference but also degrades $P_{B}$ due to the shorter transmission distance between $u_{0}$ and its serving RSU $b_{0}$. In addition, $P_{C}$ increases with both $\theta_{b}^{H}$ and $\theta_{b}^{V}$, and the introduced gain becomes significant in cases of high RSU density. This is because expanding the antenna beamwidth increases the coverage area and enlarges *t*, particularly when the transmission distance between $u_{0}$ and $b_{0}$ is small. More importantly, the vertical beam has a more significant effect on the performance of $P_{C}$ than the horizontal beam.

### 4.4. Numerical Results for Q

**Figure 8:**
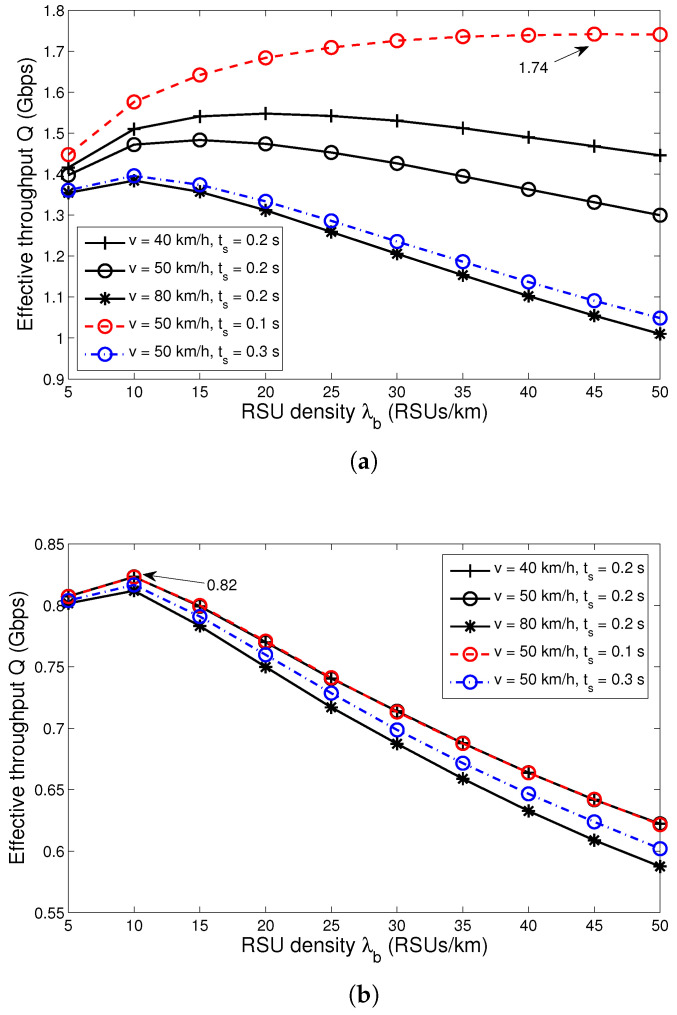
*Q* vs. $\lambda_{b}$ for various values of *v* and $t_{s}$ under (**a**) 3D beamforming and (**b**) 2D beamforming.

Figure 8 shows the results for *Q* under both 3D and 2D beamforming for various values of $\lambda_{b}$, *v*, and $t_{s}$. Simplified analytical results for *Q* under 2D beamforming [26] are shown in Figure 8b, as calculated using Corollary 3. Similar to Figure 6, *Q* increases with decreasing $t_{s}$ and *v* due to more frequent beam alignment and slower movement, resulting in higher values of $t/t_{s}$. Some important phenomena were observed when comparing Figure 8a with Figure 8b:

- *Q* first increases and then decreases when reaching the peak value as $\lambda_{b}$ becomes larger, as seen in both subfigures. The reason is that the transmission distance between $u_{0}$ and $b_{0}$ shrinks as $\lambda_{b}$ grows, and the target signal strength is enhanced; however, increasing $\lambda_{b}$ also causes more co-channel interference and a smaller *t* since the beam coverage range is reduced. When $\lambda_{b}$ is small, the former is the predominant factor, and *Q* increases with $\lambda_{b}$; when $\lambda_{b}$ is large, the latter is the predominant factor, and *Q* decreases with increased $\lambda_{b}$. Additionally, the optimal value of $\lambda_{b}$ for maximizing *Q*, decreases with increasing *v* or $t_{s}$, as shown in Figure 8a. This is because even in cases of sparse RSU deployment, a high vehicle speed or large beam alignment period would result in longer outage times, thereby degrading the value of $t/t_{s}$ and exacerbating the downward trajectory of *Q*. These phenomena suggest that an optimal RSU density that maximizes the spectral efficiency exists in 3D mmWave V2I networks, and it decreases as vehicle speed increases and the beam alignment period lengthens.
- The results for *Q* under 2D beamforming are lower than those under 3D beamforming, and the corresponding performance gaps widen as $\lambda_{b}$ increases or as *v* and $t_{s}$ decrease. This is because disregarding the vertical beam results in decreased antenna gain and increased interference due to the larger beam coverage range, leading to underestimation of the performance of *Q*. The impact of simplified 2D beamforming becomes increasingly significant as $\lambda_{b}$ increases or as *v* and $t_{s}$ decrease. When v=50 km/h and $t_{s}=0.1$ s, it is evident that the peak value of *Q* has degraded to half of that observed under 3D beamforming. These findings suggest that disregarding the vertical beam can lead to substantial underestimation of the transmission capacity, particularly in scenarios with dense RSU deployment and slow vehicles.

## 5. Conclusions

In this work, a comprehensive 3D analysis framework is established to derive the coverage and connectivity performance of a typical vehicle based on SG. Specifically, high vehicular mobility and 3D beamforming are addressed for analytical correctness. The proposed framework is evaluated through MC simulations, yielding three important findings:

1. An optimal RSU density that maximizes spectral efficiency exists, which should be set lower in higher-mobility scenarios;
2. Disregarding the effect of the vertical beam of a 3D antenna array can lead to inaccurate evaluations of coverage and connectivity performance;
3. The negative influence of high vehicular mobility on connectivity performance could be mitigated by appropriately setting the beam alignment period, RSU density, and horizontal and vertical beamwidths.

In future work, we will extend the proposed method to more realistic and complex scenarios:

1. Irregular road geometries with more realistic mobility patterns, and highly scattering urban environments;
2. UAV-V2X communication and RIS-enabled vehicular communication [40];
3. Safety-oriented advanced applications in autonomous driving with 3D V2I communication [41].

Moreover, energy efficiency and signaling overhead analyses are necessary for improving the availability and scalability of the proposed analytical framework.

## Figures and Tables

**Table 1:** Simulation parameters.

Notation	Value
$\lambda_{u}$, $\lambda_{k}$, $\lambda_{b}$	20 vehicles/km, 10 obstacles/km, 10 RSUs/km
$h_{u}$, $h_{k}$, $h_{b}$	1.6 m, 3.5 m, 10 m
$l_{k}$, $w_{k}$	12 m, 2.6 m
ρ, μ	2.2, $10^{-12.3}$
$\theta_{b}^{V}$, $\theta_{b}^{H}$, $\theta_{u}^{V}$, $\theta_{u}^{H}$	15, 30, 15, 36
*P*, $\sigma^{2}$	27 dBm, −94 dBm
$f_{c}$, *B*	28 GHz, 100 MHz
$t_{s}$, *v*	0.2 s, 80 km/h

**Figure A1:**
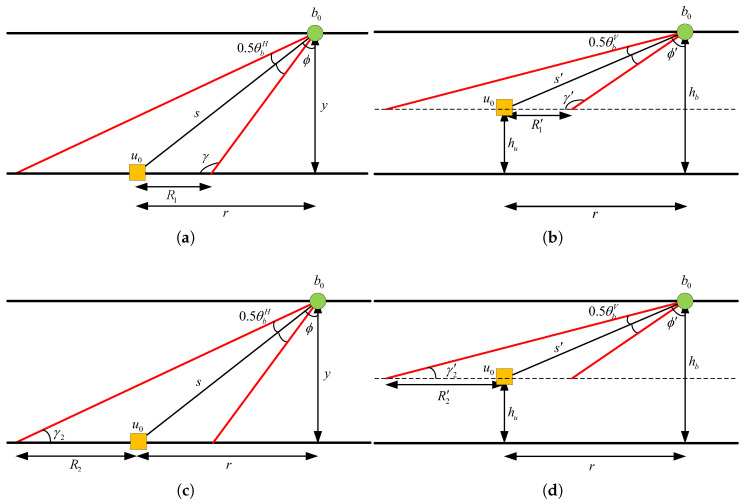
Location relationship between $u_{0}$ and $b_{0}$, and the beam coverage range of $b_{0}$ when $u_{0}$ moves toward and away from $b_{0}$. (**a**) Horizontal beam coverage of $b_{0}$ when $u_{0}$ moves toward $b_{0}$. (**b**) Vertical beam coverage of $b_{0}$ when $u_{0}$ moves toward $b_{0}$. (**c**) Horizontal beam coverage of $b_{0}$ when $u_{0}$ moves away from $b_{0}$. (**d**) Vertical beam coverage of $b_{0}$ when $u_{0}$ moves away from $b_{0}$.

## Data Availability

Data are contained within the article.

## Appendix A. Proof of Theorem 1

According to the definition of connectivity probability, we have

(A1) $$\begin{array}{cc} P_{C}\left(T\right) & =P_{\mathrm{cov}}\left(T\right)\;\cdot \;P_{B}=\int_{0}^{\infty }P\left[\mathrm{SINR}>T|r\right]P_{B}\left(r\right)f\left(r\right)dr, \end{array}$$

where the conditional coverage probability $P\left[\mathrm{SINR}>T|r\right]$ is obtained using Lemma 2, and the conditional probability of the beam sojourn, denoted by $P_{B}\left(r\right)$, can be determined through the following derivation.

First, as $u_{0}$ approaches $b_{0}$, the spatial relationships between $u_{0}$ and $b_{0}$ are depicted in Figure A1a,b. Based on the geometric configurations depicted in Figure A1a, we can mathematically define the distance $R_{1}$ from $u_{0}$ to the horizontal beam coverage edge of $b_{0}$ as

(A2) $$\begin{array}{c} R_{1}=\frac{s\sin\left(\frac{\theta_{b}^{H}}{2}\right)}{\sin(\gamma )}, \end{array}$$

where $\gamma =\frac{\pi }{2}-\frac{\theta_{b}^{H}}{2}+\arccos\left(\frac{y}{s}\right)$. Therefore, the conditional probability of $u_{0}$ existing in the horizontal beam area of $b_{0}$ throughout the beam alignment period, given the distance *r*, can be determined as

(A3) $$\begin{array}{cc} \; & P_{B}^{H}\left(r\right)=P\left[R_{1}>vt_{s}|r\right]\\ \; & =P\left[\frac{s\sin\left(\frac{\theta_{b}^{H}}{2}\right)}{\sin(\gamma )}>vt_{s}\right]\\ \; & =P\left[s>\frac{vt_{s}\left(\cos\left(\frac{\theta_{b}^{H}}{2}\right)\cos\left(\phi \right)+\sin\left(\frac{\theta_{b}^{H}}{2}\right)\sin\left(\phi \right)\right)}{\sin\left(\frac{\theta_{b}^{H}}{2}\right)}\right]\\ \; & =P\left[s>\frac{vt_{s}\left(\frac{y}{s}\cos\left(\frac{\theta_{b}^{H}}{2}\right)+\sin\left(\frac{\theta_{b}^{H}}{2}\right)\sqrt{1-\frac{y^{2}}{s^{2}}}\right)}{\sin\left(\frac{\theta_{b}^{H}}{2}\right)}\right]\\ \; & =P\left[\frac{s}{vt_{s}}-\frac{y}{s\tan(\frac{\theta_{b}^{H}}{2})}>\sqrt{1-\frac{y^{2}}{s^{2}}}\right]\\ \; & =P\left[\frac{s^{2}}{v^{2}t_{s}^{2}}+\frac{y^{2}}{s^{2}\tan^{2}\left(\frac{\theta_{b}^{H}}{2}\right)}-\frac{2y}{vt_{s}\tan\left(\frac{\theta_{b}^{H}}{2}\right)}>1-\frac{y^{2}}{s^{2}}\right]\\ \; & =P\left[\frac{z^{2}}{v^{2}t_{s}^{2}}-\left(1+\frac{2y}{vt_{s}\tan\left(\frac{\theta_{b}^{H}}{2}\right)}\right)z+y^{2}+\frac{y^{2}}{\tan^{2}\left(\frac{\theta_{b}^{H}}{2}\right)}>0\right]. \end{array}$$

By solving the inequality in (A3), we get

(A4) $$\begin{array}{c} P_{B}^{H}\left(r\right)=\left\{\begin{array}{cc} 1, & r>r_{k_{1}}^{H}\\ 0, & r\leq r_{k_{1}}^{H}, \end{array}\right. \end{array}$$

where $r_{k_{1}}^{H}$ is given by (18).

Similarly, in accordance with the geometric configuration shown in Figure A1b, we get

(A5) $$\begin{array}{c} P_{B}^{V}\left(r\right)=\left\{\begin{array}{cc} 1, & r>r_{k_{1}}^{V}\\ 0, & r\leq r_{k_{1}}^{V}, \end{array}\right. \end{array}$$

where $r_{k_{1}}^{V}$ is given by (19).

Employing Equations (A4) and (A5), the resulting representation of $P_{B}\left(r\right)$ as $u_{0}$ approaches $b_{0}$ is denoted by

(A6) $$\begin{array}{c} P_{B_{1}}\left(r\right)=\left\{\begin{array}{cc} 1, & r>\max(r_{k_{1}}^{H},r_{k_{1}}^{V})\\ 0, & r\leq \max(r_{k_{1}}^{H},r_{k_{1}}^{V}). \end{array}\right. \end{array}$$

Similarly, as $u_{0}$ moves further away from $b_{0}$, as shown in Figure A1c,d, we have

(A7) $$\begin{array}{c} P_{B_{2}}\left(r\right)=\left\{\begin{array}{cc} 1, & r>\max(r_{k_{2}}^{H},r_{k_{2}}^{V})\\ 0, & r\leq \max(r_{k_{2}}^{H},r_{k_{2}}^{V}), \end{array}\right. \end{array}$$

where the values of $r_{k_{2}}^{H}$ and $r_{k_{2}}^{V}$ are given by (18) and(19), respectively.

Due to the equal probability of two cases occurring, we obtain

(A8) $$\begin{array}{c} P_{B}\left(r\right)=\frac{P_{B_{1}}\left(r\right)+P_{B_{2}}\left(r\right)}{2}. \end{array}$$

By substituting (A8) into (A1), the result of (17) is approached.

## References

[1] Clancy J., Mullins D., Deegan B., Horgan J., Ward E., Eising C., Denny P., Jones E., Glavin M. (2024). Wireless Access for V2X Communications: Research, Challenges and Opportunities. IEEE Commun. Surv. Tutor..

[2] Song Y., Xiao Y., Liu J. (2025). Enabling Adaptive Optimization of Energy Efficiency and Quality of Service in NR-V2X Communications via Multiagent Deep Reinforcement Learning. IEEE Internet Things J..

[3] Deng H., Li H., Tian Z., Tian J., Du W. (2026). Precise Time Synchronization in Packet Networks Using Deep Learning for Future Intelligent Transportation. Sensors.

[4] Chen R., Sun S., Liu Y., Hu X., Hui Y., Cheng N. (2024). Proactive Effects of C-V2X-Based Vehicle-Infrastructure Cooperation on the Stability of Heterogeneous Traffic Flow. IEEE Internet Things J..

[5] Aoki S., Yonezawa T., Kawaguchi N., Steenkiste P., Rajkumar R. (2022). Time-Sensitive Cooperative Perception for Real-Time Data Sharing over Vehicular Communications: Overview, Challenges, and Future Directions. IEEE Internet Things Mag..

[6] Yang Q., Fu S., Wang H., Fang H. (2021). Machine-Learning-Enabled Cooperative Perception for Connected Autonomous Vehicles: Challenges and Opportunities. IEEE Netw..

[7] Lucas-Estan M., Coll-Perales B., Shimizu T., Gozalvez J., Higuchi T., Avedisov S., Altintas O., Sepulcre M. (2023). Direct-V2X Support with 5G Network-Based Communications: Performance, Challenges and Solutions. IEEE Netw..

[8] Noor-A-Rahim M., Liu Z., Lee H., Khyam M.O., He J., Pesch D., Moessner K., Saad W., Poor H.V. (2022). 6G for Vehicle-to-Everything (V2X) Communications: Enabling Technologies, Challenges, and Opportunities. Proc. IEEE.

[9] Saad M.M., Khan M.T.R., Shah S.H.A., Kim D. (2021). Advancements in Vehicular Communication Technologies: C-V2X and NR-V2X Comparison. IEEE Commun. Mag..

[10] Tan J., Luan T.H., Guan W., Wang Y., Peng H., Zhang Y., Zhao D., Lu N. (2024). Beam Alignment in mmWave V2X Communications: A Survey. IEEE Commun. Surv. Tutor..

[11] 3GPP (2019). Study on NR Vehicle-to-Everything (V2X). Technical Report TR 38.885 v16.0.0.

[12] Mollah M.B., Wang H., Karim M.A., Fang H. (2024). mmWave Enabled Connected Autonomous Vehicles: A Use Case With V2V Cooperative Perception. IEEE Netw..

[13] Saluja D., Singh R., Saluja N., Kumar S. (2023). Connectivity Improvement of Hybrid Millimeter Wave and Microwave Vehicular Networks. IEEE Trans. Intell. Transp. Syst..

[14] Li J., Niu Y., Wu H., Ai B., Chen S., Feng Z., Zhong Z., Wang N. (2022). Mobility Support for Millimeter Wave Communications: Opportunities and Challenges. IEEE Commun. Surv. Tutor..

[15] Kose A., Lee H., Foh C., Dianati M. (2021). Beam-Based Mobility Management in 5G Millimetre Wave V2X Communications: A Survey and Outlook. IEEE Open J. Intell. Transp. Syst..

[16] Zhong W. (2024). Image-Based Beam Tracking With Deep Learning for mmWave V2I Communication Systems. IEEE Trans. Intell. Transp. Syst..

[17] Ding H., Shin K.G. (2023). Context-Aware Beam Tracking for 5G mmWave V2I Communications. IEEE Trans. Mob. Comput..

[18] Kim J., Chung H., Kim I., Noh G. (2024). Integration of 5G mmWave-Enabled V2I and V2V: Experimental Evaluation. IEEE Commun. Mag..

[19] Zhao Y., Zhang X., Gao X., Yang K., Xiong Z., Han Z. (2024). LSTM-Based Predictive mmWave Beam Tracking via Sub-6 GHz Channels for V2I Communications. IEEE Trans. Commun..

[20] Tassi A., Egan M., Piechocki R.J., Nix A. (2017). Modeling and Design of Millimeter-Wave Networks for Highway Vehicular Communication. IEEE Trans. Veh. Technol..

[21] Wang Y., Venugopal K., Molisch A.F., Heath R.W. (2018). MmWave Vehicle-to-Infrastructure Communication: Analysis of Urban Microcellular Networks. IEEE Trans. Veh. Technol..

[22] Yi W., Liu Y., Deng Y., Nallanathan A., Heath R.W. (2019). Modeling and Analysis of MmWave V2X Networks With Vehicular Platoon Systems. IEEE J. Sel. Areas Commun..

[23] Wu P., Li X., Zheng H., Wang K., Qin J., Tang M. (2023). 3D Modeling and Analysis of Cooperative Perception-Oriented Millimeter-Wave V2I Networks With Information Value-Based Relay. IEEE Trans. Veh. Technol..

[24] Choi C.S., Baccelli F. (2022). A Stochastic Geometry Model for Spatially Correlated Blockage in Vehicular Networks. IEEE Internet Things J..

[25] Choi C.S., Kim J., Choi J. (2025). Stochastic Geometry Analysis of RIS-Assisted Cellular Networks with Reflective Intelligent Surfaces on Roads. IEEE Trans. Commun..

[26] Giordani M., Rebato M., Zanella A., Zorzi M. (2018). Coverage and Connectivity Analysis of Millimeter Wave Vehicular Networks. Ad Hoc Netw..

[27] Bafqi S.F., Yazdi Z.Z., Asadi A. (2021). Analytical Framework for Mmwave-Enabled V2X Caching. IEEE Trans. Veh. Technol..

[28] Aghashahi S., Aghashahi S., Zeinalpour-Yazdi Z., Tadaion A., Asadi A. (2023). Stochastic Modeling of Beam Management in mmWave Vehicular Networks. IEEE Trans. Mob. Comput..

[29] Shafie A., Yang N., Durrani S., Zhou X., Han C., Juntti M. (2021). Coverage Analysis for 3D Terahertz Communication Systems. IEEE J. Sel. Areas Commun..

[30] Huawei, HiSilicon UL/DL beam management for latency/overhead reduction, 2019. Proceedings of the 3GPP TSG RAN WG1 Meeting R1-1903974.

[31] Yan D., Guan K., He D., Ai B., Li Z., Kim J., Chung H., Zhong Z. (2020). Channel Characterization for Vehicle-to-Infrastructure Communications in Millimeter-Wave Band. IEEE Access.

[32] Jameel F., Wyne S., Nawaz S.J., Chang Z. (2019). Propagation Channels for mmWave Vehicular Communications: State-of-the-art and Future Research Directions. IEEE Wirel. Commun..

[33] Kovalchukov R., Moltchanov D., Samuylov A., Ometov A., Andreev S., Koucheryavy Y., Samouylov K. (2019). Evaluating SIR in 3D Millimeter-Wave Deployments: Direct Modeling and Feasible Approximations. IEEE Trans. Wirel. Commun..

[34] Wang Y., Venugopal K., Molisch A.F., Heath R.W. Blockage and Coverage Analysis with MmWave Cross Street BSs Near Urban Intersections. Proceedings of the IEEE International Conference on Communications (ICC).

[35] Wu S., Atat R., Mastronarde N., Liu L. (2018). Improving the Coverage and Spectral Efficiency of Millimeter-Wave Cellular Networks Using Device-to-Device Relays. IEEE Trans. Commun..

[36] Weber S., Andrews J.G., Jindal N. (2010). An Overview of the Transmission Capacity of Wireless Networks. IEEE Trans. Commun..

[37] Zheng H., Wu P., Cao L. Throughput Analysis for Dynamic Millimeter-Wave V2I Networks with 3D Beamforming. Proceedings of the IEEE International Conference on Communications (ICC).

[38] 3GPP (2019). Study on evaluation methodology of new Vehicle-to-Everything (V2X) use cases for LTE and NR. Technical Report TR 37.885 v15.3.0.

[39] 3GPP (2019). Study on channel model for frequencies from 0.5 to 100 GHz. Technical Report TR 38.901 v16.1.0.

[40] Arif M., Kim W. (2024). Clustered Jamming in U-V2X Communications with 3D Antenna Beam-width Fluctuations. Comput. Commun..

[41] Wiseman Y., Grinberg I. Circumspectly Crash of Autonomous Vehicles. Proceedings of the 2016 IEEE International Conference on Electro Information Technology (EIT).

